# Chronic disturbance in the thalamus following cranial irradiation to the developing mouse brain

**DOI:** 10.1038/s41598-019-45973-8

**Published:** 2019-07-03

**Authors:** Martina Boström, Yohanna Eriksson, Jolie Danial, Thomas Björk-Eriksson, Marie Kalm

**Affiliations:** 1 Department of Pharmacology, Institute of Neuroscience and Physiology at the Sahlgrenska Academy, University of Gothenburg, Gothenburg, Sweden; 2 Department of Oncology, Institute of Clinical Sciences at the Sahlgrenska Academy, University of Gothenburg, Gothenburg, Sweden

**Keywords:** Astrocyte, Cellular neuroscience, Molecular neuroscience

## Abstract

Better survival rates among pediatric brain tumor patients have resulted in an increased awareness of late side effects that commonly appear following cancer treatment. Radiation-induced changes in hippocampus and white matter are well described, but do not explain the full range of neurological late effects in childhood cancer survivors. The aim of this study was to investigate thalamus following cranial irradiation (CIR) to the developing brain. At postnatal day 14, male mice pups received a single dose of 8 Gy CIR. Cellular effects in thalamus were assessed using immunohistochemistry 4 months after CIR. Interestingly, the density of neurons decreased with 35% (*p* = 0.0431) and the density of astrocytes increased with 44% (*p* = 0.011). To investigate thalamic astrocytes, S100β^+^ cells were isolated by fluorescence-activated cell sorting and genetically profiled using next-generation sequencing. The phenotypical characterization indicated a disrupted function, such as downregulated microtubules’ function, higher metabolic activity, immature phenotype and degraded ECM. The current study provides novel insight into that thalamus, just like hippocampus and white matter, is severely affected by CIR. This knowledge is of importance to understand the late effects seen in pediatric brain tumor survivors and can be used to give them the best suitable care.

## Introduction

Better survival rates among pediatric brain tumor patients have resulted in an increased awareness of the late side effects that can appear following cancer treatment. The treatment strategies used to cure a child from a brain tumor consist of surgery, chemotherapy and radiotherapy. It is well known that radiotherapy towards the brain can cause lifelong late effects, such as impairments in processing speed, working memory, executive function and attention^1,2^. Further, it has been shown that even low doses of ionizing radiation to the brain can cause intellectual impairment as well as perturbed growth and puberty^3^. The mechanisms of chronic radiation damage have been proposed to be related to long-term toxicity of oligodendrocytes, endothelial cells and normal neural cell types, including stem and progenitor cells, in combination with metabolic derangements, glial reactions, and inflammatory responses^2,4^. Most research have focused on radiation-induced changes in the hippocampus and white matter^1,5^, but injuries in those brain regions do not explain the full range of all neurological late effects seen in childhood cancer survivors.

Cranial radiotherapy during childhood has been coupled with altered sleep-wake rhythm in adulthood^6^. A brain region important for this control is the thalamus^7,8^. However, the role of the thalamus in pediatric brain tumor survivors remains to be clarified. The thalamus is located between the cortex and the midbrain with direct connectivity to many subcortical structures, and was formerly believed to be merely a passive central sensory and motor relay station. Today, there is evidence implicating that the thalamus is contributing directly to cognitive functions as diverse as memory, attention, perception, motor planning, language processing, motivation, decision-making, and the monitoring of self-generated actions^7^. At the same time, the specific mechanisms by which the thalamus shapes these cognitions and behaviors remain unknown. Due to thalamus’ role in functions that are greatly affected by cranial radiotherapy (e.g. sleep/wakefulness and cognition), and the lack of research performed within the area, it poses a highly interesting brain region to investigate further following cranial radiotherapy.

The aim of this study was to investigate chronic alterations in the thalamus 4 months following cranial irradiation (CIR) to the young mouse brain. Cellular effects in the thalamus was first assessed by stereological quantifications of neurons, microglia, oligodendrocytes and astrocytes. These analyses revealed a decreased density of neurons and an increased density of astrocytes in the thalamus following CIR. To further unravel the role of this increased proportion of astrocytes, S100 calcium-binding protein (S100β)^+^ astrocytes were isolated from the thalamus by fluorescence-activated cell sorting (FACS) and genetically profiled using next-generation sequencing (NGS). This phenotypical characterization indicated that thalamic astrocytes are affected by CIR with a disrupted function, such as downregulated microtubules’ function, higher metabolic activity, immature phenotype and degraded ECM. This study provides novel insight into that the thalamus, just like the hippocampus and white matter, is severely affected by CIR.

## Results

### Cellular composition in the thalamus following CIR

**Figure 1:**
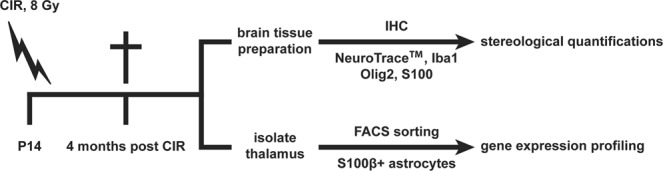
An overview of the study outline. At postnatal day 14 (P14), the animals were subjected to a single dose of 8 Gy cranial irradiation (CIR). The animals were sacrificed 4 months later. One group of animals was used for immunohistochemistry (IHC) to quantify neurons (NeuroTrace^TM^), Iba1^+^ microglia, Olig2^+^ oligodendrocytes and S100^+^ astrocytes in the thalamus. Another group of animals was used to isolate S100β^+^ thalamic astrocytes using fluorescence-activated cell sorting (FACS) and perform gene expression profiling using next-generation sequencing (NGS).

In the current study, postnatal day 14 (P14) mice were subjected to a single dose of 8 Gy CIR and sacrificed 4 months later. The study outline is illustrated in Fig. 1. Cellular composition in the thalamus following CIR was examined by stereological quantifications (thoroughly described in the method section).

#### Volume

**Figure 2:**
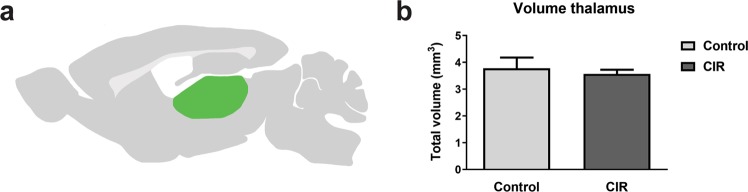
Volume measurement of the thalamus. An illustration of the location of the thalamus (green) in the mouse brain is shown in (**a**). The volume of the thalamus was measured 4 months following CIR (**b**). Data shown as mean ± SEM. CIR = cranial irradiation, n = 10–11 per group.

We have previously observed a smaller volume in both the hippocampus and the corpus callosum following CIR^9,10^. Therefore, the volume was measured to assess the CIR-induced injury in the thalamus. However, the thalamic volume was not affected by CIR in the current study (Fig. 2b).

#### Neurons

**Figure 3:**
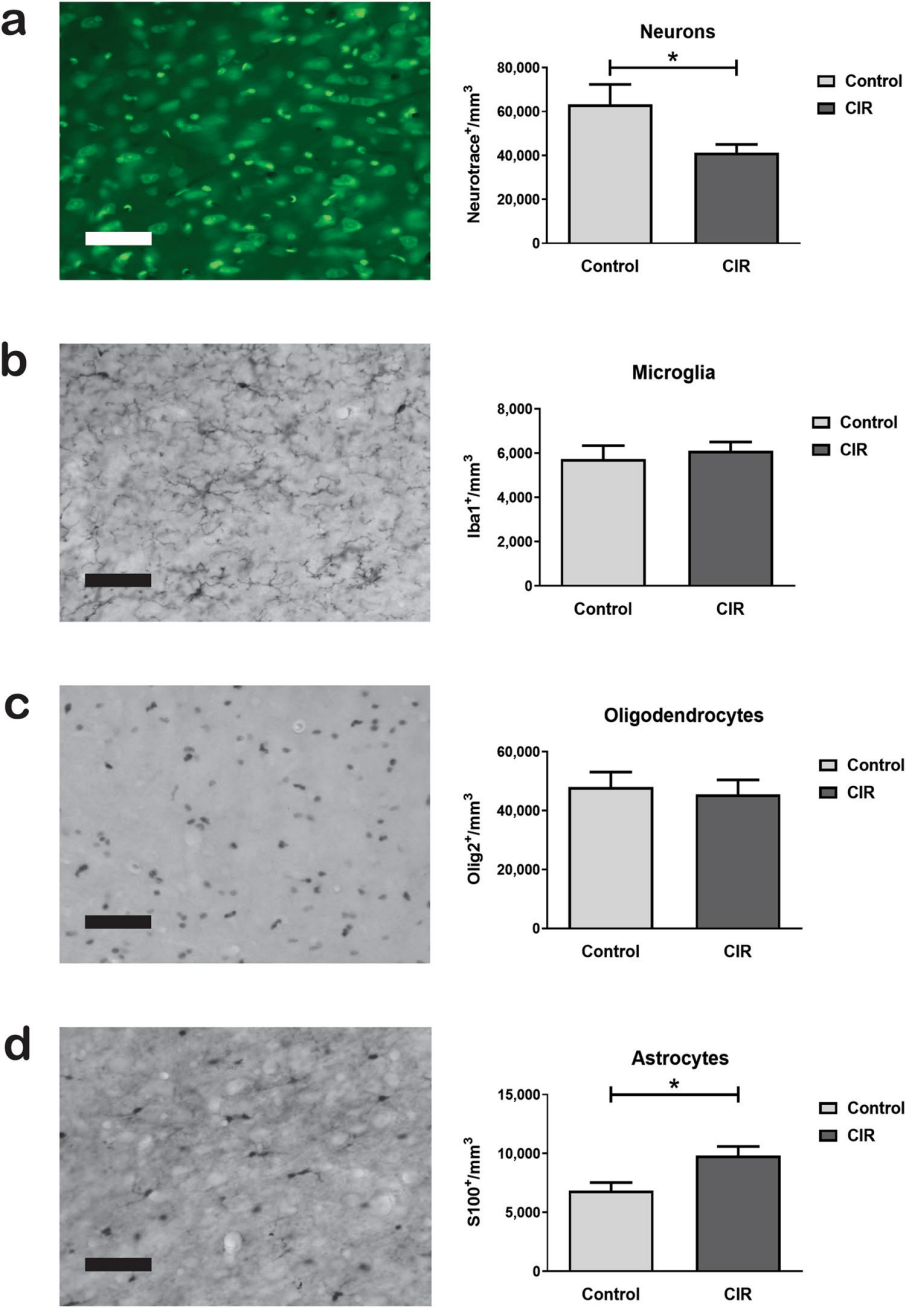
Cellular effects in the thalamus following CIR. Cellular quantifications were performed using a stereological approach in the thalamus 4 months following CIR at P14. A representative microphotograph and the density of mature neurons (NeuroTrace^TM^) (**a**), Iba1^+^ microglia (**b**), Olig2^+^ oligodendrocytes (**c**), and S100^+^ astrocytes (**d**) are shown. *p < 0.05. Data shown as mean ± SEM. CIR = Cranial irradiation, P14 = postnatal day 14, n = 9–11 per group. Scale bar = 50 µm.

Cellular effects in the thalamus was further assessed by quantifying mature neurons (NeuroTrace^TM^). Interestingly, the density of thalamic neurons was reduced with 35% in irradiated animals compared to controls (*p* = 0.0431, Fig. 3a).

#### Microglia

To further evaluate non-neuronal cells we quantified ionized calcium-binding adapter molecule 1 (Iba1)^+^ microglia in the thalamus. Microglia are known to play a key function in the inflammatory response in the brain and loss of these cells have previously been shown in the juvenile brain following CIR^11^. However, their response in the thalamus has remained unexplored. The thalamic microglia were unchanged 4 months following CIR-induced injury to the developing brain (Fig. 3b).

#### Oligodendrocytes

We have previously observed a radiation-induced reduction of oligodendrocytes in the hippocampus (12 days following CIR) and in the corpus callosum (4 months following CIR)^9,10^. Considering the important function in generating myelin sheets to facilitate neuronal signaling within the central nervous system (CNS), we decided to quantify oligodendrocyte transcription factor 2 (Olig2)^+^ oligodendrocytes in the thalamus. However, in contrast to the hippocampus and the corpus callosum, no difference was observed in the density of oligodendrocytes 4 months after CIR in the thalamus (Fig. 3c).

#### Astrocytes

Astrocytes are another non-neuronal cell population that have a key role in the healthy brain, but also following an injury. We have previously observed effects on astrocytes in the hippocampus as long as one year following CIR^12^. In the current study, we performed stereological quantifications of S100^+^ astrocytes (possibly including a subpopulation of neurons). Interestingly, the density of astrocytes was increased by 44% (p = 0.011, Fig. 3d). This was a particularly interesting finding considering that the opposite effect was seen in the density of neurons.

### Gene expression in astrocytes following CIR

**Figure 4:**
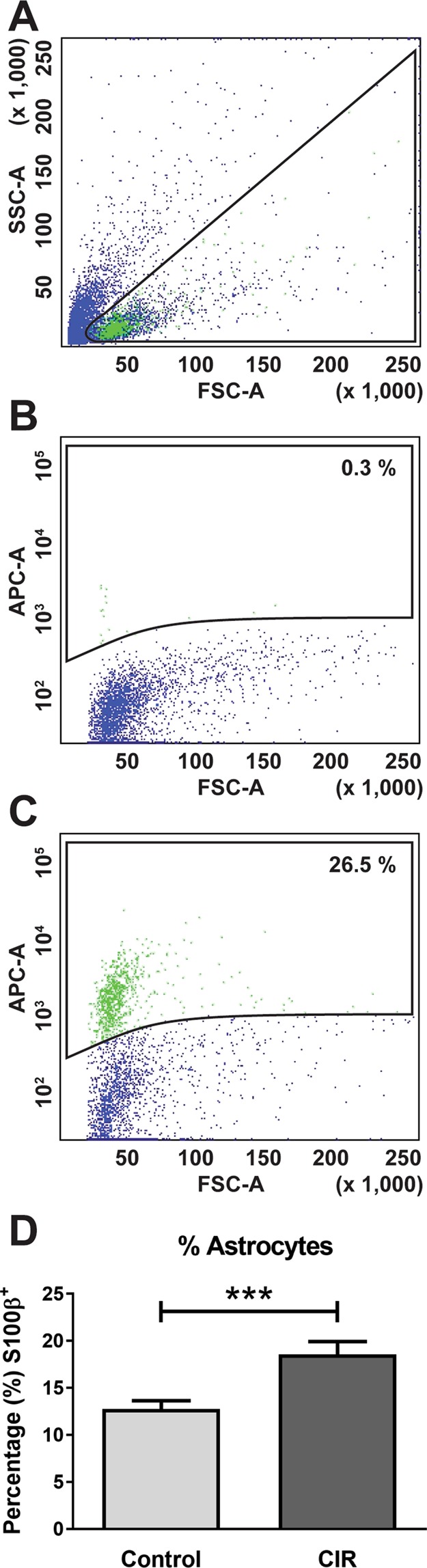
FACS analysis of S100β^+^ astrocytes in the brain following CIR. Gating strategy for sorting of S100β^+^ astrocytes from the thalamus. (**A**) shows the SSC vs FSC plot which was used to gate cells from debris, (**B**) shows a negative control staining where the primary antibody was omitted and the (**C**) panel shows a representative sample stained with S100β. (**D**) show quantification of the relative numbers (%) of S100β^+^ astrocytes within the entire heterogeneous cell population analyzed by flow cytometry during the isolation of astrocytes with FACS. ***p < 0.001. Data shown as mean ± SEM. CIR = Cranial irradiation, FACS = fluorescence-activated cell sorting, FSC = forward scatter, SSC = side scatter, n_control_ = 20 and n_irradiated_ = 12.

**Figure 5:**
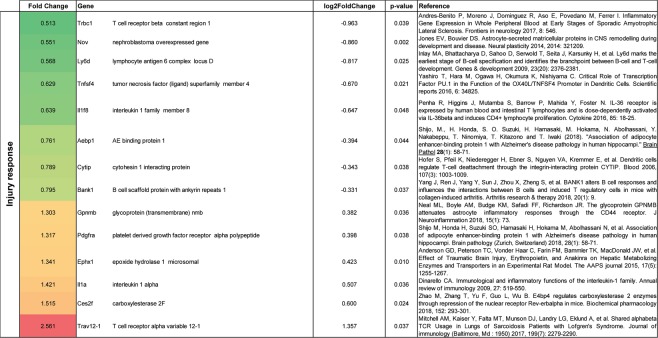
Gene alterations related to injury-responses in thalamic astrocytes following CIR. Significantly altered genes are shown. Genes were evaluated on PubMed and the most relevant reference were added. Data are presented as relative gene expression compared to controls in log2 scale. CIR = cranial irradiation.

**Figure 6:**
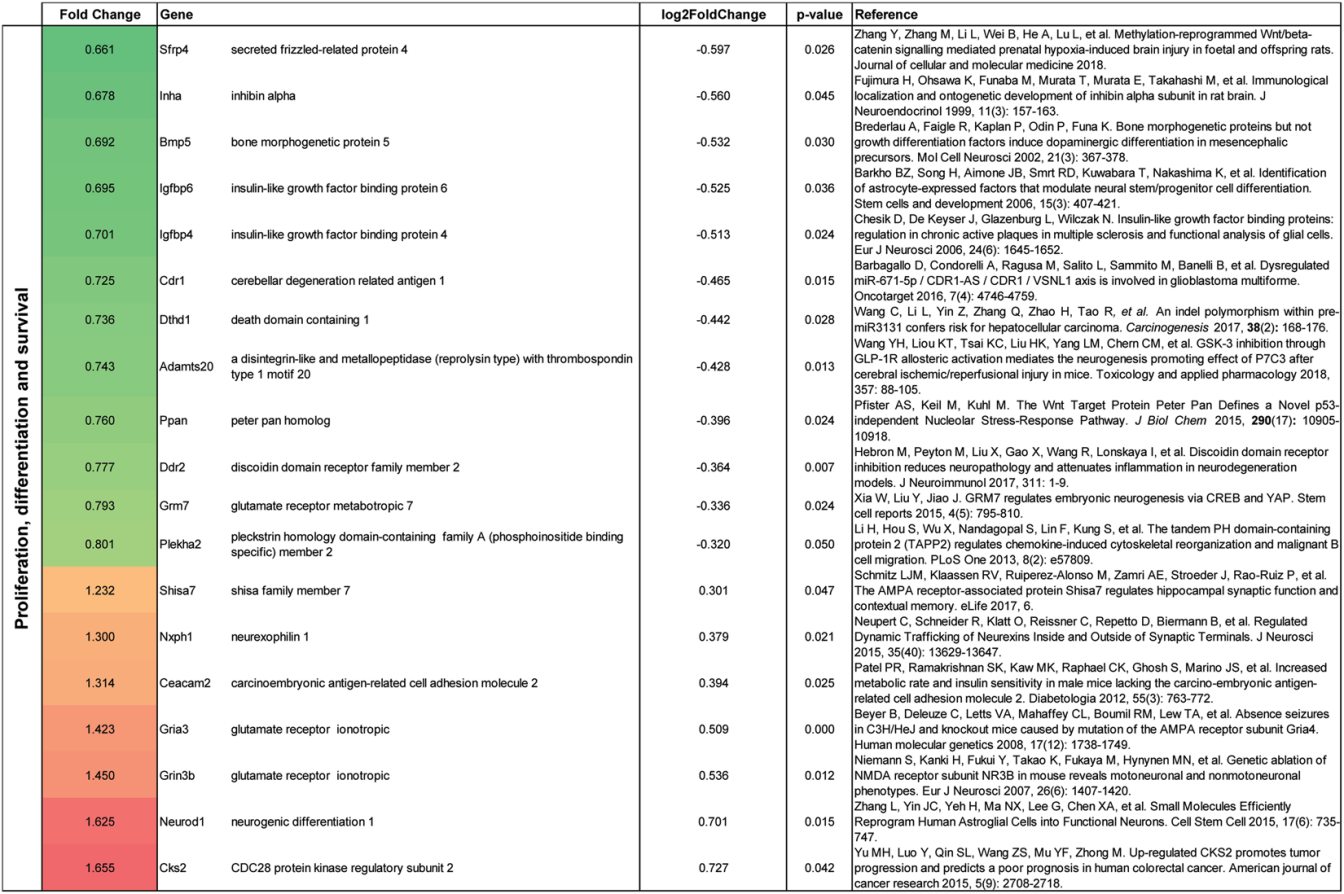
Gene alterations related to proliferation, differentiation, and survival in thalamic astrocytes following CIR. Significantly altered genes are shown. Genes were evaluated on PubMed and the most relevant reference were added. Data are presented as relative gene expression compared to controls in log2 scale. CIR = cranial irradiation.

**Figure 7:**
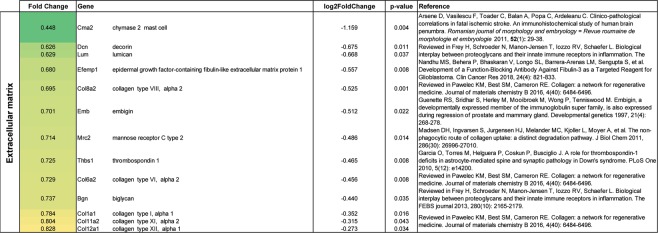
Gene alterations related to extracellular matrix in thalamic astrocytes following CIR. Significantly altered genes are shown. Genes were evaluated on PubMed and the most relevant reference were added. Data are presented as relative gene expression compared to controls in log2 scale. CIR = cranial irradiation.

**Figure 8:**
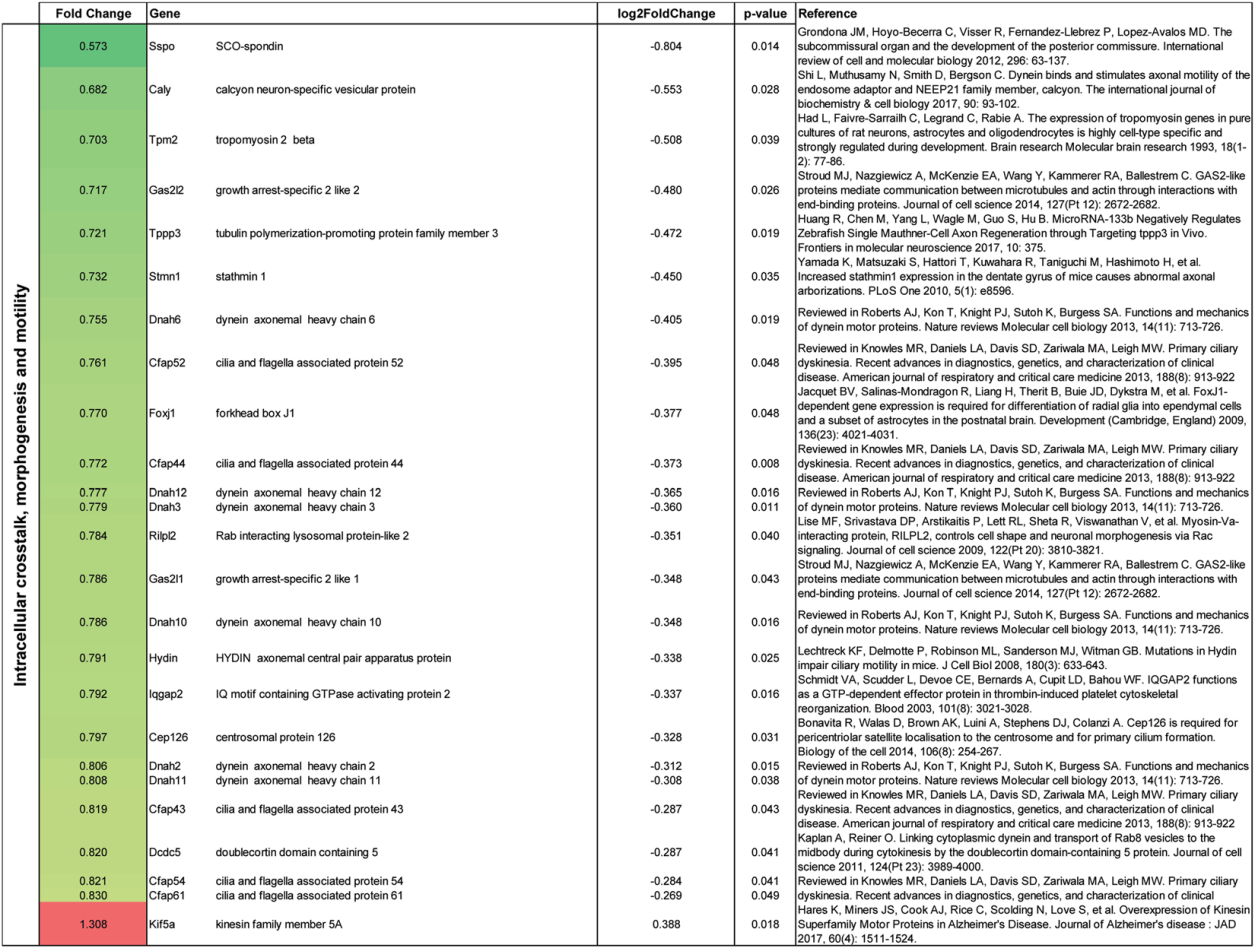
Gene alterations related to intracellular crosstalk, morphogenesis and motility following CIR. Significantly altered genes are shown. Genes were evaluated on PubMed and the most relevant reference were added. Data are presented as relative gene expression compared to controls in log2 scale. CIR = cranial irradiation.

To further unravel the role of thalamic astrocytes in radiation-induced injury, we chose to isolate S100β^+^ astrocytes by FACS (Fig. 4A–C) and phenotypically characterize those using NGS. The flow cytometry analysis showed an increased amount of astrocytes by 54% in CIR-treated animals compared to controls (*p* < 0.001, Fig. 4D), similar to the stereologically assessed quantification (44% increase). Further, the NGS analysis showed a CIR-induced transcriptome change in thalamic astrocytes. Of the significantly altered genes, 57 were up-regulated and 155 were down-regulated after CIR (Supplementary Table 1). Genes that were found to be annotated or predicted genes on GeneCards^®^ (https://www.genecards.org/), were further evaluated on PubMed. Those described in the literature, and that were related to the following irradiation-induced injury mechanism: injury response; proliferation, differentiation and survival; extracellular matrix; intracellular crosstalk, morphogenesis and motility, are described in more detail below (Figs 5–8).

Fourteen injury-related genes were affected following CIR (Fig. 5), where eight were downregulated and six were upregulated. The thalamic astrocytes are presumably set to a healing state, with activated clearance mechanisms and lower levels of inflammation, as indicated by increased levels of *Nov*, *Gpnmb, Ces2f* and *Ephx1*^13–16^. However, some genes (*Bank1, Il1a*, and *Pdgfra*) indicate that there is still an ongoing injury present as long as 4 months following CIR^17–19^.

Among the genes that were related to proliferation, differentiation and survival, 12 genes were downregulated and seven were upregulated (Fig. 6). The increased expression of *Ceacam2* suggests a higher metabolic activity in astrocytes following CIR^20^. This could possibly be explained by the increased expression of genes involved in proliferation, e.g. *Cks2*^21^. Further, genes related to astrocytic development (*Inha* and *Bmp5*) indicate an immature phenotype^22,23^. In line with this, the astrocytes attempt to restore the lack of neurons as indicated by increased levels of *Neurod1*, which has been shown to be able to convert astrocytes to neurons^24^. In general, genes involved in synaptic plasticity and behavior, propose that the astrocytes are trying to restore the CIR-induced dysfunction (*Sfrp4, Plekha2, Nxph1*)^25–27^.

Following prophylactic radiotherapy to the brain, lower levels of extracellular matrix (ECM) proteins in the cerebrospinal fluid from adult small cell lung cancer patients have previously been shown^28^. In line with this, all genes related to ECM showed reduced expression in the current study, indicating a degradation of the ECM following CIR (Fig. 7). This could play an important part in the healing process following CIR, since the ECM function as a barrier for plasticity and axon regeneration.

Among the genes that were related to intracellular crosstalk, morphogenesis and motility, 24 genes were downregulated and one was upregulated following CIR (Fig. 8). Genes important for microtubules’ function were all downregulated (*Stmn1*, *Tppp3, Dnah2*, *Dnah3*, *Dnah6*, *Dnah10*, *Dnah11*, *Dnah12, Gas2l1* and *Gas2l2*)^29–31^. Further, genes involved in actin reorganization and stabilization were all downregulated (*Tpm2, Iqgap2*)^32,33^. However, *Kif5a*, connected to transport of cellular organelles and proteins^34^ was upregulated. Taken together, the gene expression following CIR indicates a disturbed astrocytic phenotype in the thalamus.

## Discussion

The survival rate for pediatric brain tumor patients has improved significantly and advancements in the health care will most likely lead to a continuously increasing number of survivors in the society, living with treatment-induced side effects that could have a negative impact on their quality of life. In this study, we wanted to increase the knowledge regarding the lifelong late effects seen following cranial radiotherapy to the developing brain with focus on the thalamus. The main findings from this study were the following: 1) The density of neurons were decreased in the thalamus after CIR. 2) The amount of astrocytes were increased in the thalamus following CIR. 3) The phenotype of the astrocytes are affected by CIR, with alterations such as downregulated microtubules’ function, higher metabolic activity, immature phenotype and degraded ECM, indicating a disrupted function. 4) The volume of the thalamus was not altered by CIR to the developing brain. This knowledge adds another piece to the puzzle in the complexity behind brain regions that are affected by cranial radiotherapy.

In this study the density of neurons in the thalamus was decreased, whereas the density of astrocytes was increased. This cellular disturbance most likely interferes with the normal function of the thalamus. A similar cellular effect following cranial radiation has been shown in the hippocampus, where inflammation has been suggested as the driving force in making precursor cells become astrocytes instead of neurons^35^. Interestingly, the thalamus display similar alterations as the hippocampus except for the lack of growth previously seen in the hippocampus^36,37^, and no effects on oligodendrocytes and microglia. In the hippocampus, both microglia and oligodendrocytes are decreased following irradiation to the young mouse brain^10,11^. This is an interesting difference between the brain regions, which could possibly be explained by that the thalamus at the time of CIR (P14) is more mature compared to the hippocampus, making the thalamus less vulnerable. For example, the mediodorsal thalamus in rodents has its growth peak before P13^38^ whereas hippocampus is still growing at the time of CIR^36,37^. Differences between brain regions following CIR have previously been shown in a study where the subventricular zone recovered with time but the hippocampus did not^39^. In our previous studies of white matter and the hippocampus, a decrease of oligodendrocytes was observed^9,10^. The current study show a different response with unaltered density of oligodendrocytes but less neurons in the thalamus, indicating that the oligodendrocytes have less axons to myelinate. Oligodendrocytes are known to be able to myelinate several axons simultaneously^40^. This study is the first to report these observed cellular disturbances in the thalamus following CIR-induced damage.

The increased density of astrocytes was shown using two different methods (immunohistochemistry and FACS), thereby strengthening the validity of the data. It has previously been shown that quantification of cell populations using flow cytometry correlate well with quantitative immunohistochemistry^41^. Our NGS results from the FACS isolated astrocytes indicate that the astrocytes are trying to restore the CIR-induced dysfunction (*Sfrp4, Plekha2, Nxph1*)^25–27^ and even try to convert into neurons (*Neurod1*)^24^ (Fig. 6). For example, *Sfrp4*, known to inhibit the Wnt pathway that controls cell proliferation, differentiation and migration, was downregulated following CIR^25^. The gene expression data do however also indicate that there is an ongoing injury (*Bank1, Il1a*, and *Pdgfra*)^17–19^ (Fig. 5), and all genes related to ECM were downregulated (Fig. 7). To illustrate, *Il1a*, a proinflammatory cytokine with costimulatory effects of different T lymphocyte functions, was increased following CIR by 42%^18^. However, it should be noted that the vast down-regulated gene expression observed in thalamic astrocytes in the current study (57 were up-regulated and 155 were down-regulated after CIR), could be a consequence of the increased numbers of thalamic astrocytes in irradiated animals. In this study, an increased expression of *Ceacam2* (Fig. 6) was observed, suggesting a higher metabolic activity in thalamic astrocytes following CIR^20^. This finding is supported by a study of adult male macaque monkeys which showed increased glucose metabolism in the thalamus following a total dose of 40 Gy fractionated whole-brain irradiation (fWBI)^42^. Together, these results indicate that as long as 4 months following CIR to the young brain there are still ongoing processes related to the CIR-induced injury. It remains to determine which of these processes that poses a target of interest to restore normal brain function following CIR. The results from the astrocytic gene expression analysis should however be interpreted with precaution as many of the genes have previously not been associated with or shown to be expressed by astrocytes. Another aspect to keep in mind is that type of radiation, dose rate, and single or fractionated dosing could affect the radiobiological response. For example, a study performed on adult rats propose that the cellular response following a single dose of cranial irradiation does not predict the cellular response following biological equivalent fractioned radiation doses^43^. Further, it has been shown that the molecular response to fractionated irradiation is slower compared to single dose irradiation^44,45^. It is also important to note that the adult and the juvenile brain respond significantly different to cranial irradiation, at least in the hippocampus^46^. In summary, the data presented in the present study provide novel insight into the unexplored function of astrocytes in the thalamus, both in naïve and irradiated animals.

Late effects following cranial radiotherapy in childhood, such as altered sleep-wake rhythm and problems with memory and attention, could all be linked to the thalamus due to the diverse role of this brain region^6–8^. In this study, we did not observe any change in volume of the thalamus after CIR, which could explain why the thalamus has not been studied in greater detail following radiotherapy towards the brain. For example, both white matter and the hippocampus are known to display smaller volumes following cranial radiotherapy and both, especially the hippocampus, are well described in the literature^1,9,10,47–49^. However, the volumetric results from the thalamus in the current study are supported by a previous study from our group, as well as a study of children with acute lymphoblastic leukemia that also showed no change in thalamic volume following cranial radiotherapy^10,50^. Regarding cellular alterations in the thalamus following cranial radiotherapy, one study has shown reduced gray matter density in the thalamus after prophylactic cranial irradiation to adult small cell lung cancer patients^51^. Moreover, another study showed higher apparent diffusion coefficient (ADC) values, measured by diffusion tensor imaging (DTI), in the thalamus of children with medulloblastoma treated with craniospinal radiation compared to controls^52^. An increased ADC indicate that there are more free water in the tissue following radiation, which is of particular interest since the NGS data from the current study point towards a degraded ECM. More water in the tissue could also explain the reduced gray matter density from the Simo *et al*. study. Further, Mabbot *et al*., showed that decreased fractional anisotropy (FA) and increased ADC were related to lower intellectual outcome in patients relative to age-matched controls^52^. In another study, FA values were significantly reduced in normal appearing cerebral white matter of the temporal lobe, hippocampus, and thalamus in adult survivors treated with fWBI for acute lymphoblastic leukemia^53,54^. The correlation between compromised fiber integrity, tissue loss, and lower IQ is consistent with findings in previous studies that show that white matter volume loss is related to adverse intelligence and academic outcome^55,56^. Our findings shed light on why the clinical DTI data from the thalamus are altered following cranial radiotherapy, and show that the thalamus can be added to the list of affected regions in the brain following cranial radiotherapy.

The current study revealed an increased density of astrocytes and decreased density of neurons, a disturbance that is most likely contributing to the late effects seen in pediatric brain tumor survivors. These results now need to be verified further in humans and the question of how to restore the thalamus still remains. Several studies have used pharmacological interventions and physical exercise following CIR to the developing brain and investigated effects in for example the hippocampus^10,57,58^. It would now be of great interest to see if any of them also have positive effects in the thalamus following CIR-induced damage. The new knowledge presented in the current study is of importance to understand the late effects seen in pediatric brain tumor survivors and can be used in the future to give them the best suitable habilitation.

## Materials and Methods

### Animals

Male C57BL/6J mice were purchased from Charles River Breeding Laboratories (Sulzfeld, Germany. Animals were housed according to normal procedures at the Experimental Biomedicine animal facility (University of Gothenburg, Gothenburg, Sweden). The mice were kept on a 12-h light cycle with food and water provided *ad libitum*. The room temperature was 19–21 °C with 40–70% relative humidity. All experiments were approved by the Swedish Animal Welfare Agency (application no. 2015–72). Further, all experiments were performed in accordance with relevant guidelines and regulations.

### Irradiation procedure

A linear accelerator (Varian Clinac 1600 C-2 (250 Mu/min, 2.4 Gy/min) and True Beam STX (600 Mu/min, 5.6 Gy/min), Radiation Oncology Systems, LLC, San Diego, CA, USA) with 6 MV nominal photon energy were used to irradiate mice on P14. The mice were anesthetized with an intraperitoneal (i.p.) injection of tribromoethanol (Sigma, Stockholm, Sweden), placed on a polystyrene bed in prone position (head to gantry) and irradiated with a symmetrical 2 × 2 cm radiation field. A tissue equivalent material covered the head to obtain an even radiation dose distribution in the underlying tissue. The source to skin distance was approximately 99.5 cm and the irradiated tissue received a single absorbed dose of 8 Gy with a dose variation of ±5%. Using the LQ model^59^ and an alpha/beta ratio of 3 for late effects in the normal brain tissue, the acute exposure of 8 Gy is equivalent to approximately 18 Gy when delivered in repeated 2 Gy fractions. Animals were kept on a warm bed (36 °C) both before and after CIR to maintain body temperature. Control animals were anesthetized but did not receive any CIR.

### Cellular composition in the thalamus following CIR

#### Tissue preparation

Animals were deeply anesthetized with sodium pentobarbital (Pentothal, Electra-box Pharma, Tyresö, Sweden) before being transcardially perfused with a 6% formaldehyde solution buffered with sodium phosphate at pH 7.4 and stabilized with methanol (Histofix; Histolab products AB, Gothenburg, Sweden). The brains were immersion-fixed in Histofix for 24 h after perfusion and then changed to 30% sucrose solution containing 100 mM phosphate buffer, pH 7.5. After equilibration in sucrose, the brains were fixed with a cryo-gel (Tissue-Tec^®^ O.C.T. compound, Sakura^®^ Finetek, Alphen aan den Rijn, The Netherlands) to a dry ice-cooled copper block and one hemisphere was sagitally cut into 25 μm sections with a sliding microtome (Leica SM2000R, Leica Microsystems, Nussloch, Germany). Serial sections were collected in a series of 12 tubes containing tissue cryoprotectant solution (TCS; 25% ethylene glycol, 25% glycerine and 0.05 M phosphate buffer), and stored at 4 °C until used for immunohistochemistry.

#### NeuroTrace^TM^ Fluorescent Nissl stain

Sections were rinsed in TBS and then washed in TBS with 0.1% Triton X-100 for 10 min. After further washing, sections were incubated with NeuroTrace^TM^ 500/525Green Fluorescent Nissl stain for 20 min (N21480, ThermoFisher Scientific, USA). Following several rinsing steps in TBS, the sections were mounted and cover slipped with ProLong® Gold Antifade Reagent (Thermo-Fisher Scientific, USA).

#### Immunohistochemistry for Iba1, Olig2 and S100

Stainings for stereological analysis were performed on every 12th section and rinsed in Tris-buffered saline (TBS; 0.08 mol/L Trizma-HCl, 0.016 mol/L Trizma-Base, 0.15 mol/L NaCl, pH 7.5) before staining. After rinsing in TBS, sections underwent two subsequent steps to avoid unspecific binding. First, endogenous peroxidase activity was blocked by incubating the sections in 30% hydrogen peroxide (H_2_O_2_) for 30 min, followed by rinsing in TBS. This was followed by 30 min incubation in TBS with 3% donkey serum and 0.1% Triton X-100 (blocking solution) to avoid unspecific antigen binding. Sections were incubated at 4 °C overnight with primary antibodies against Iba1 (rabbit anti-Iba1, 1:1000, Wako Pure Chemical Industries Ltd. Osaka, Japan), Olig2 (goat anti-Olig2, 1:1000, R&D Systems, Minneapolis, MN,USA) or S100 (rabbit anti-S100, 1:5000, Dako Cytomation, Glostrup, Denmark), diluted in blocking solution. The following day, the sections were rinsed and a biotinylated secondary antibody was added for 1 h at room temperature (donkey anti-rabbit IgG, 1:1000, or donkey anti-goat IgG, 1:1000, all from Jackson Immunoresearch Laboratories Inc). This was followed by rinsing and amplification with avidin-biotin enzyme complex (ABC kit, Vectastain Elite, Vector Laboratories, Burlingame, CA, USA) for 1 hour. Finally, sections were once again rinsed and staining was developed using 0.25 mg/mL 3–30-diaminobenzindine tetrahydrochloride (DAB, Saveen Werner AB, Malmö, Sweden) diluted in TBS containing 0.009% H_2_O_2_ and 0.04% nickel chloride to enhance the reaction. Omission of the primary antibodies yielded only very weak nonspecific staining, and the identification of the immunopositive cells was facilitated by their characteristic morphology and location.

#### Stereological procedures

Cells were counted in every 12^th^ section using systematic-random sampling (Stereoinvestigator, MicroBrightField, USA) and a Leica DM6000 B microscope (Leica Microsystems, Germany) at 20x magnification. Counting started on sections containing a clearly separated/divided dorsal and ventral hippocampus. The thalamic area was traced at either 5x or 10x magnification. Total volumes were calculated according to the Cavalieri principle, using the following formula: V = SA × P × T, where V is the total volume, SA is the sum of area measurements, P is the inverse of the sampling fraction and T is the section thickness. For Iba1, all immunopositive cells in the thalamus were counted. The total number of Iba1^+^ cells was obtained by multiplying the number of counted cells with the series fraction (1/12), and the density acquired by dividing the total number of Iba1^+^ cells with the volume.

For neurons (NeuroTrace^TM^ staining), Olig2^+^ and S100^+^ cells, a grid (500 × 500-μm for NeuroTrace^TM^, 375 × 375-μm for Olig2 and 300 × 300-μm for S100) was randomly placed over the thalamic traced area and counting frames (100 × 100-μm for NeuroTrace^TM^ and Olig2, and 150 × 150-μm for S100) were placed within the grid. The total number of cells per animal was calculated by dividing the number of counted cells with the sampling fractions, i.e., fraction of sampling area/total traced area × series fraction (1/12) × optical dissector height/physical section thickness. Finally, the density was calculated by dividing the total number of cells with the volume.

### Next generation sequencing of isolated astrocytes

#### Isolation of cells from tissue

Mice were deeply anesthetized with sodium pentobarbital (Pentothal^®^; Electra-box Pharma, Tyresö, Sweden) and decapitated 4 months after CIR. Brains were rapidly removed from the cranium, followed by rinsing in sterile 0.9% NaCl. The thalamus was collected by microdissection and transferred into gentleMACS C Tubes (Milentyi Biotec, Bergisch Gladbach, Germany) containing 2 mL of ice cold papain/protease/DNase I enzyme solution consisting of 0.01% papain (Worthington, Lakewood, NJ, USA), 0.1% Dispase II (SigmaAldrich, Saint Louis, MO, USA), 0.01% DNase I (Worthington, Lakewood, NJ, USA) and 12.4 mM MgSO_4_ diluted in HBSS without Ca^2+^ and Mg^2+^ (Hank’s Balanced Salt Solution; Worthington, Lakewood, NJ, USA). The left and right thalamus from the same animal were placed in a tube as one single sample and further processed together according to Milentyi Biotec´s adult brain dissociation kit for mice and rat (with significant modification), in order to obtain a single cell suspension devoid of connective tissue.

GentleMACS C tubes were tightly closed and attached upside down onto the sleeve of the gentleMACS Octo Dissociator. The gentleMACS **m_brain_01** program was run, followed by 15 min incubation on low rotation at 37 °C. This was followed by running the **m_brain_02** program, 10 min incubation on low rotation at 37 °C, running the **m_brain_03** program and finally 10 min incubation on low rotation at 37 °C. The C tubes were thereafter centrifuged briefly to collect the sample at the bottom of the tube. Samples were triturated ~15 times (in about 30 s) with a cotton plugged 9 inch pasteur pipette with the tip barely fire polished to an opening of 0.7–1.1 mm D (inferior) (ORIGIO, Målov, Denmark), followed by 10 min incubation at 37 °C. After incubation, tissue pieces were once again gently triturated ~15 times, followed by 10 min incubation at 37 °C and finally gently triturated ~15 times until no pieces were visible.

MACS SmartStrainers (70 µm) were placed on 15 mL Falcon tubes and washed with ~10 mL of cold (4 °C) Dulbecco´s Phosphate-buffered Saline (D-PBS; Gibco/Invitrogen, San Diego, CA, USA). The fluid was discarded. Resuspended samples were applied to the MACS SmartStrainer, followed by rinsing the C tubes with 5 mL cold (4 °C) D-PBS and transferring the fluid onto the MACS SmartStrainers. An additional 5 mL cold (4 °C) D-PBS was then applied onto the MACS SmartStrainers before they were discarded. Cell suspensions in the 15 mL Falcon tubes were centrifuged at 100 × g for 10 min at 4 °C and supernatant were aspirated completely. The cell suspensions were resuspended in 1 mL of D-PBS and transferred to 2 mL Eppendorf tubes.

#### Debris removal

A volume of 300 µL cold Debris Removal Solution (Milentyi Biotec, Bergisch Gladbach, Germany) were added to the cell suspension and mixed thoroughly. The mixed solution was gently overlaid with ~700 µL cold D-PBS, making sure that the phases were not mixed. After 10 min centrifugation at 200 × g at 4 °C, three phases were formed, and the two upper phases were completely aspirated and discarded. The tubes were filled up with cold D-PBS to a final volume of 2 mL and gently inverted 3 times.

#### Fixation and staining of cells before flow cytometry

Immediately after isolation, the cells were fixed and stained as previously described^41^, with some modifications. The cell suspension was centrifuged at 200 × g for 10 min at 4 °C and the supernatant aspirated before fixation for 15 min in 200 µL Fix and Perm^®^ Fixation Buffer A (Invitrogen/Life Technologies, Carlsbad, CA, USA). Cells were washed with 1 mL Stain Buffer (FBS) (BD Biosciences Pharmigen, Franklin Lakes, NJ, USA), centrifuged at 400 × g for 10 min at 4 °C and the supernatant aspirated. This was followed by incubation for 20 min with primary antibody against S100β (monoclonal mouse anti-bovine S100β, 1:250, LifeSpan BioSciences Inc./Nordic BioSite, Täby, Sweden) diluted in 200 µL Fix and Perm^®^ Permeabilization Buffer B (Invitrogen/Life Technologies, Carlsbad, CA, USA). After another wash with Stain Buffer (as above), the cells were incubated for 20 min in the dark with the secondary antibody diluted 1:1000 in 200 µL Fix and Perm^®^ Permeabilization Buffer B (donkey anti-mouse Cy5, Jackson Immunoresearch Laboratories, West Grove, PA, USA). Finally, the cells were washed with Stain Buffer, resuspended in 400 µL of Stain Buffer and transferred to FACS tubes.

#### Isolation of S100^+^ astrocytes with fluorescence-activated cell sorting

S100^+^ astrocytes were isolated from the thalamus at 4 months post-CIR using a FACS Aria II equipped with a 100 µm nozzle and DIVA software (BD Biosciences Pharmigen, Franklin Lakes, NJ, USA). Samples were progressively gated based first on size (FSC: forward scatter; size) and granularity (SSC: side scatter; granularity), followed by a pulse geometry gate (FSC-H x FSC-A) where doublets were excluded from the analysis. As controls, amorphous secondary gates were based on cells stained with the secondary antibody only. The cells were sorted with FACS Flow Sheath Fluid (BD Biosciences Pharmigen, Franklin Lakes, NJ, USA) directly into 2 mL sterile micro tubes. Total cell number per sorted population was recorded, resulting in an average of 12,739 ± 746 and 27,630 ± 1,888 astrocytes for controls and irradiated, respectively. Representative data was captured for the first 10,000 cells.

#### RNA isolation

Immediately after the isolation of cells with FACS, the cell suspension was centrifuged for 20 min at 13,400 rpm, the supernatant was completely aspirated and the cells were frozen at −80 °C until further processed. Total RNA from sorted cells was purified using the RNeasy kit for formalin-fixed paraffin-embedded (FFPE) tissue sections according to the protocol provided by the manufacturer (Qiagen, Hilden, Germany). In order to isolate enough RNA for next generation sequencing, 2–3 animals were pooled into one sample. For controls: 20 animals were pooled into 7 samples, and for irradiated: 12 animals were pooled into 6 samples. Total RNA was eluted in 15 µL RNase-free water and RNA concentrations were measured using a NanoDrop Spectrophotometer (Thermo Scientific, Wilmington, DE, USA).

#### Next generation sequencing

RNA integrity was assessed using the TapeStation RNA ScreenTape (Agilient Technologies, SantaClara, CA, USA). The samples had low RNA integrity number (RIN) values (between 2-2,7) and were degraded. In the current study we used Illumina’s TruSeq^®^ Total Stranded RNA kit with Ribo-Zero (Gold) (Illumina, San Diego, CA, USA) and optimized some of the steps from the standard protocol to achieve an optimal result. TruSeq^®^ Total Stranded RNA kit with Ribo-Zero (Gold) protocol is based on ribosomal RNA depletion and is suitable for degraded RNA samples.

A total of 10 μL (∼100 ng) from each sample were used for starting the library preparation. Directly after depletion, a cleanup was performed using 200 uL of the RNAClean XP beads (Beckman Coulter, Brea, CA, USA) for each sample. The fragmentation step was not performed and samples were directly continued to the first strand synthesis of cDNA step. Further, the PCR cycle was increased to 18 cycles for all samples. Libraries were quantified and normalized with Qubit DNA HS Assay kit (Invitrogen/Life Technologies, Carlsbad, CA, USA) and TapeStation (Agilent Technologies, Santa Clara, CA, USA), as recommended by Illumina. The libraries prepared using the same protocol were pooled together. Libraries were sequenced on a NextSeq. 500 with a High output reagent (150 cycles) with a read length of 2 × 75 bp at the Genomics Core Facility, at the Sahlgrenska Academy, University of Gothenburg.

#### Bioinformatic analysis

The quality of the data was evaluated using Fastqc (http://www.bioinformatics.babraham.ac.uk/projects/fastqc) and the fastq files were filtered using prinseq (version 0.20.3)^60^. Further, the quality filtered fastq files were mapped towards the mouse reference genome (mm10, UCSC assembly, 2011) using STAR (version 2.5.2b)^61^. The resulting alignment was indexed using SAMtools (version 1.3.1)^62^. Moreover, overall mapping quality was examined with qualimap (version 2.2.1)^63^ and the gene counts were calculated with HTseq (version 0.5.3p3)^64^. The differential expression was computed using DESeq2^65^. Finally, a pathway analysis was performed on the significantly expressed genes (*P*-value 0.05) with IPA (Ingenuity^®^ Systems, http://www.ingenuity.com/).

### Statistical analysis

Cellular quantifications were analyzed using an unpaired Student’s t-test, where *p* < 0.05 was considered statistically significant (GraphPad Prism 5.00). All data are shown as mean ± S.E.M. Statistical analysis for NGS is described above.

## Supplementary information

Supplementary table 1

## Data Availability

The datasets generated during and/or analysed during the current study are available from the corresponding author on reasonable request.
